# Hepatitis B Vaccination Reliability in Celiac Disease

**DOI:** 10.5812/kowsar.1735143X.761

**Published:** 2011-08-01

**Authors:** Mohammad Rostami Nejad, Kamran Rostami, Mohammad Reza Zali

**Affiliations:** 1 Research Center for Gastroenterology and Liver Disease, Shahid Beheshti University of Medical Sciences, Tehran, Iran; 2 Acute Medicine, Dudley Group of Hospital, Dudley, UK

**Keywords:** Hepatitis B virus, Vaccination, Antibody

Vaccination is an efficient and reliable means of protection against common hepatitis B virus (HBV) infections [1]. Between 90% and 95% of the adult population responds to HBV vaccination [2][3], and 4-10 % of vaccine recipients fail to respond to standard immunization [1][4][5]. The ability to respond to recombinant HBV vaccine is associated with immunogenetically condition because of multiple candidate genes [6][7]. Some specific HLA haplotypes are considered to be the most important genetic markers for non-responders, who are found to carry specific haplotypes such as B8, DR3, and DQ2 [6][7]. Since HLA genotypes play an important role in unresponsiveness to the HBV vaccine, celiac disease (CD) may be associated with this unresponsiveness [8]. CD is a common autoimmune disorder, which has a particularly strong association with the presence of HLA-DQ2 in 90-95% of the patients [9][10][11][12].

In an issue of Hepatitis Monthly [13], Ertekin et al. reported the results of their study on 52 children with CD and 20 age- and sex-matched healthy children who received HBV vaccination as per an immunization schedule. The proportion of children in the CD group who failed to respond to HBV vaccination (32 of 52) was significantly higher than the proportion of control individuals who did not (18 of 20; 61.5% vs. 90%; P < 0.05). Ertekin et al. concluded that unresponsiveness to hepatitis B vaccination was found in a higher percentage in children with CD than the control group. They concluded that response to the HBV vaccine in children with CD should be investigated and a different immunization schedule should be developed for them. They also suggested that compliance with a gluten-free diet may improve the immune response of celiac children to the HBV vaccine. Most studies on this topic have demonstrated that the number of children with CD who failed to respond to the HBV vaccine is significantly greater than that of healthy children [4][5][8][14].

In a similar study on an adult cohort, Noh et al. found that of 23 adults with CD who had completed a full course of HBV vaccination, 19 tested positive for HBsAb and 13 did not show long-term immunity [15].

This association of HLA with non-responsiveness to HBV vaccination was further confirmed in a study by Stachowski et al., where 34 out of 153 patients with end-stage renal disease were non-responsive to a recombinant HBV vaccine and HLA-DQ2 was found almost exclusively in the non-responder group [7].

Longer intervals between vaccination and antibody testing might be one of the reasons for significantly low protective post-vaccination HBV antibody titers even in CD patients who comply with dietary guidelines [8][14]. Therefore, revaccination is recommended when the patients are following a controlled gluten-free diet.

This study was not designed to determine the presence of HLA-DQ2 and HLA-DQ8 in both the groups. Therefore, future studies evaluating the HLA haplotypes in CD and control groups should aim to characterize the role of HLA typing in the response to HBV vaccination.

As in the case of other conditions and as indicated by the strong evidence for the protective role of GFD, early diagnosis of CD may obviously increase the percentage of patients responding to the HBV vaccine. Moreover, beginning with a short duration, strict, gluten-free diet seems to play a positive role in the development of antibody memory. Given the high prevalence of CD in the general population and a lack of response to HBV vaccine in untreated patients, we think that non-responsiveness to HBV vaccine necessitates routine assessment in patients with CD. Non-responsiveness to HBV vaccine may be a possible sign of undiagnosed CD or may suggest noncompliance with gluten-free diet.
